# Circulating IL-1*β*, IL-17, and IP-10 as Potential Predictors of Hepatitis B Virus Infection Prognosis

**DOI:** 10.1155/2022/5202898

**Published:** 2022-06-22

**Authors:** Zhenzhen Su, Jie Chen, Junlong Zhang, Yunfei An, Yun Liao, Xiaojuan Wu, Chuanmin Tao, Lanlan Wang, Bei Cai

**Affiliations:** Department of Laboratory Medicine/Research Centre of Clinical Laboratory Medicine, West China Hospital of Sichuan University, Chengdu 610041, China

## Abstract

Circulating cytokines and chemokines play critical roles in hepatitis B virus (HBV) infection. Here, we explored the effects of proinflammatory and anti-inflammatory effector molecules on HBV progression, e antigen seroconversion, and liver function. Our results showed that circulating interleukin (IL)-17 may be helpful in HBV spontaneous clearance [odds ratio (OR) = 1.468, 95%confidence interval (CI) = 1.080–1.995, *P* = 0.014] and protective against HBV-related hepatoma development (OR = 0.933, 95%CI = 0.910–0.957, *P* < 0.001). IL-1*β* negatively affected HBV clearance (OR = 0.052, 95%CI = 0.005–0.534, *P* = 0.013). In patients with chronic hepatitis B, interferon-*γ*-inducible protein-10 (IP-10) levels significantly increased in the group of abnormal liver function (*P* = 0.006). Furthermore, positive correlations of IP-10 with alanine aminotransferase and aspartate aminotransferase levels were observed (*r*_*s*_ = 0.546 and 0.644, respectively; *P* < 0.001). In conclusion, inflammatory cytokines and chemokines may be a “double-edged sword” for HBV clearance and progression. Further exploration of the roles of IL-17, IL-1*β*, and IP-10 in chronic HBV infection is needed.

## 1. Introduction

Hepatitis B virus (HBV) infection is a global health problem that is particularly pronounced in China. As is currently known, chronic inflammation in patients with HBV increases the average risk of carcinogenesis over time. Alterations in immune system function play a critical role in the progression and prognosis of HBV. Recent evidence suggests that HBV may not directly cause liver inflammation, hepatocellular injury, and liver cancer but that these diseases are induced by the immune reaction to HBV, which is mainly caused by the disruption of cellular immune function [1, 2].

Cells of the immune system exert not only cytotoxic effects but also supporting effects, mainly by releasing molecular mediators of immune functions, namely, cytokines and chemokines. Inflammatory and anti-inflammatory cytokines, as well as chemokines, play a critical role in chronic inflammation associated with HBV [3, 4], wherein serum interleukin (IL)-1*β*, IL-6, and IL-17 are the crucial proinflammatory cytokines. IL-10, an important anti-inflammatory cytokine, plays an immunosuppressive role in fibrogenesis. Interferon-*γ*-inducible protein-10 (IP-10/CXCL10), monocyte chemoattractant protein-1 (MCP-1/CCL2), macrophage inflammatory protein-1*β* (MIP-1*β*/CCL4), and IL-8 (IL-8/CXCL8) are chemokines that induce the migration of macrophages, monocytes, neutrophil granulocytes, and lymphocytes towards inflamed tissue, thus exerting their effects [5]. Therefore, cytokines and chemokines are critical effector molecules during inflammatory injury in their own characteristic ways.

Most studies exploring cytokine effects on hepatitis C virus (HCV) progression have been performed from the perspective of identifying genetic polymorphisms and measuring protein expression levels [6, 7]. These results indicate that increased inflammatory responses induced by cytokines and chemokine signaling are present in patients with HCV. However, studies on the roles of cytokines and chemokines in HBV infection are limited. Several researchers have focused on the association between cytokine expression and the status of HBeAg and liver inflammation in patients with chronic hepatitis B (CHB) [8, 9]. Here, we aimed to obtain a better understanding of the effects of cytokine/chemokine on chronic HBV infection and explore biomarkers that can predict HBV prognosis.

## 2. Materials and Methods

### 2.1. Subjects

A total of 123 patients with chronic HBV infection and 70 patients with HBV spontaneous clearance (SC, patients with natural clearance of HBsAg) were recruited from West China Hospital of Sichuan University. Patients with chronic HBV infection were further classified into CHB (*N* = 81) and HBV-related hepatoma (*N* = 42) groups based on clinical diagnosis. Patients with CHB had at least 6 months of HBsAg positivity and anti-HBs negativity and had no evidence of decompensated cirrhosis or carcinoma. Meanwhile, patients with hepatoma who had HBsAg positivity were diagnosed using histopathology, imaging, and/or laboratory testing. HBV SC subjects were negative for HBsAg, but positive for anti-HBs and anti-HBc; only individuals without a vaccination history confirmed via face-to-face interviews were recruited. All subjects were classified into normal liver function (NLF) and abnormal liver function (ANLF) groups. The criteria of NLF were as follows: total bilirubin (TB) ≤28.0 *μ*mol/L; alanine aminotransferase (ALT) ≤60 IU/L in male and ≤50 IU/L in female; and aspartate aminotransferase (AST) ≤55 IU/L in male and ≤50 IU/L in female. All other conditions were identified as ANLF. In addition, all patients infected with HBV were also classified into the low HBV DNA load (HBV − DNA ≤ 1 × 10^5^ IU/mL) and high HBV-DNA load (HBV − DNA > 1 × 10^5^ IU/mL) groups. The exclusion criteria were as follows: patients with hepatitis A, C, or D virus or human immunodeficiency virus infection and those with alcoholic liver disease, chronic liver disease due to other causes, acute viral hepatitis B, and treatment with drugs other than nucleos(t)ide analogs.

Twenty-nine healthy individuals were enrolled as the controls. All subjects in the control group had no infectious or autoimmune diseases and did not have tumors at the time of enrolment. All healthy individuals had normal liver function. Our study was performed in accordance with the current revision of the Helsinki Declaration and approved by the West China Hospital Ethics Committee. Written informed consent was obtained from all the participants. The clinical characteristics for all the subjects are listed in Table 1.

### 2.2. Measurement of Circulating Cytokines and Chemokines

Circulating levels of cytokines, including IL-1*β*, IL-6, IL-17, and IL-10, and chemokines, including IL-8, IP-10, MCP-1, and MIP-1*β*, were measured using the Bio-Plex system and Bio-Plex Pro™ human cytokine reagent kits (Bio-Rad, Hercules, CA, USA), according to the manufacturer's instructions.

### 2.3. HBV Viral Load, Serology, and Biochemical Assays

Serum HBV DNA was extracted using the NucliSENS easyMAG system (Biomerieux Company, Paris, France), and the viral load was measured using the Roche Light Cycler 480 II (Roche Diagnostics, Basel, Switzerland) according to the manufacturer's instructions.

Serological markers of HBV, including HBsAg, HBeAg, anti-HBs, anti-HBe, and anti-HBc, were analyzed using the Elecsys Modular E170 immunoassay (Roche Diagnostics, GmbH, Mannheim, Germany). Clinical biochemical analysis of TB, direct bilirubin (DB), indirect bilirubin (IB), ALT, AST, albumin (ALB), alkaline phosphatase (ALP), gamma-glutamyl transferase (GGT), and total protein (TP) was conducted using the Cobas c702 assay (Roche Diagnostic, GmbH, Mannheim, Germany) via photocolorimetry. Red blood cell (RBC), white blood cell (WBC), and platelet (PLT) counts of the whole blood were analyzed using a Sysmex XE 5000 (Sysmex, Kobe, Japan).

### 2.4. Statistics

All data were analyzed using SPSS (version 25.0; IBM, Armonk, NY, USA) and GraphPad Prism 9.0 (GraphPad Software, San Diego, CA, USA). Data with normal distribution were described as mean ± standard deviation and analyzed with ANOVA or Student's *t*-test to make comparisons among the groups. Meanwhile, data with nonnormal distribution were described as median (interquartile) and analyzed with Kruskal-Wallis H or Mann–Whitney *U* tests to make group comparisons, as well as made Spearman correlation analysis. Categorical data were analyzed using *χ*^2^ or Fisher's exact test. A multivariate logistic regression model based on the forward LR stepwise method was used to evaluate the factors affecting HBV infection outcomes. *P* < 0.05 was considered to be significant difference.

## 3. Results

### 3.1. Characteristics of the Enrolled Subjects

As shown in Table 1, no significant differences in age and sex distribution were observed among the groups; most of the enrolled subjects were male (62.07%–71.43%). HBeAg positivity (69.14% vs. 21.43%) and high HBV DNA load (3.59 × 10^6^ IU/mL vs. 1.00 × 10^3^ IU/mL) were more common in the CHB group compared to that in the hepatoma group. Except for IB (*P* = 0.229) and PLT (*P* = 0.888), significant differences in liver function indicators were observed among the groups (*P* < 0.05).

### 3.2. Association of Circulating Cytokine and Chemokine Levels with HBV Infection Outcomes

**Figure 1:**
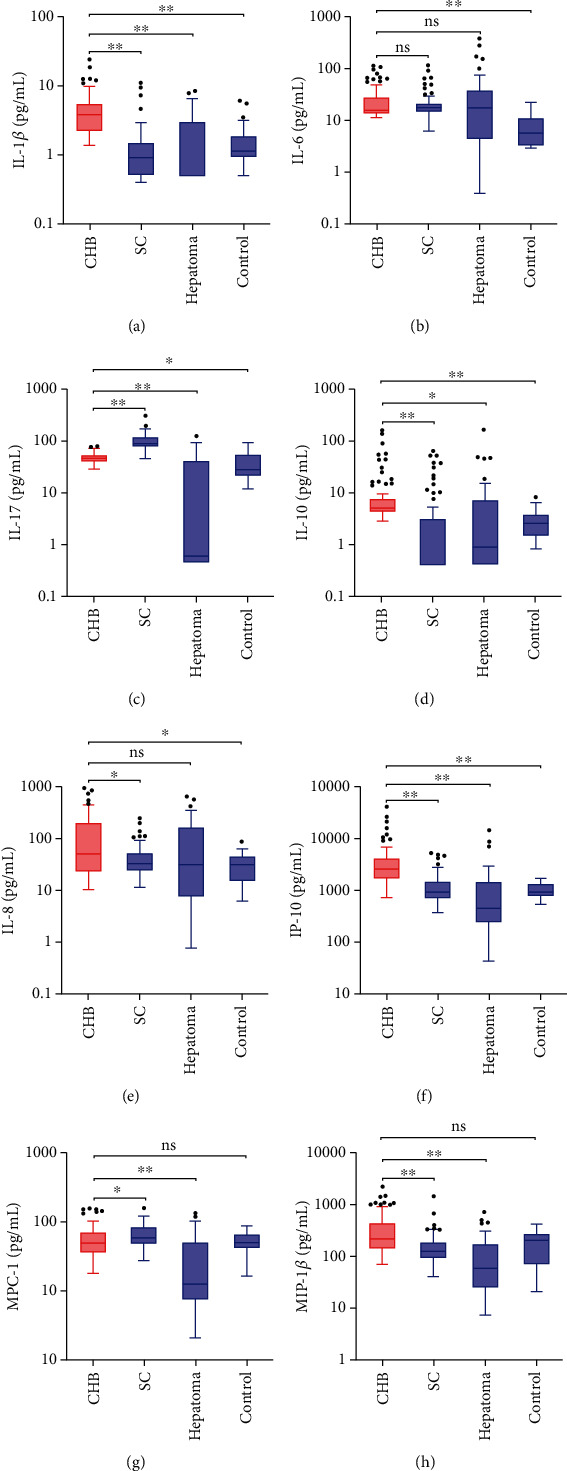
Circulating cytokine and chemokine levels in patients with chronic hepatitis B (CHB), HBV spontaneous clearance (SC), and HBV-related hepatoma and in healthy controls. It shows circulating cytokine IL-1*β* (a), IL-6 (b), IL-17 (c), and IL-10 (d) and chemokine IL-8 (e), IP-10 (f), MCP-1 (g), and MIP-1*β* (h) levels in different groups. Each box plot represents the median, interquartile range, and the minimum and maximum values. The Mann–Whitney *U* test was used to compare the CHB (red) and other groups (blue), and Bonferroni correction was applied to adjust for multiple comparisons. ^∗^*P* value <0.05, and ^∗∗^*P* value <0.001. ns: Not significant.

The levels of the analyzed circulating cytokines and chemokines were significantly different among the CHB, SC, hepatoma, and control groups (Table 2). Except for IL-6, the distribution of all cytokines/chemokines was significantly different between the CHB and SC groups. IL-1*β*, IL-10, IL-8, IP-10, and MIP-1*β* levels were higher in the CHB group, whereas IL-17 and MCP-1 levels were higher in the SC group. Considering HBV progression, the levels of IL-1*β*, IL-17, IL-10, IP-10, MCP-1, and MIP-1*β* were all markedly decreased in the hepatoma group compared to that in the CHB group (Table 2, Figure 1).

Furthermore, multivariable logistic regression analysis included cytokines and chemokines that were significantly different between the groups when using univariate analysis (Table 3). IL-17 appeared to be helpful in HBV clearance [SC group, odds ratio (OR) = 1.468, 95%confidence interval (CI) = 1.080–1.995, *P* = 0.014] and protective against hepatoma development (hepatoma group, OR = 0.933, 95%CI = 0.910–0.957, *P* < 0.001). Meanwhile, IL-1*β* negatively affected HBV clearance (SC, OR = 0.052, 95%CI = 0.005–0.534, *P* = 0.013).

### 3.3. Association of Circulating Cytokine and Chemokine Levels with HBeAg Status, HBV DNA Load, and Liver Function

**Figure 2:**
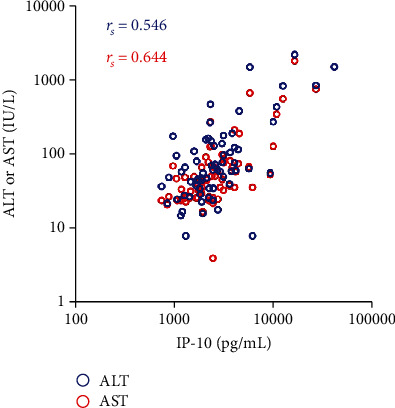
Correlation of the circulating IP-10 with ALT and AST concentrations in chronic hepatitis B patients. Correlations were assessed using the Spearman correlation coefficient (*r*_*s*_).

Patients with CHB were divided into different groups according to their clinical characteristics. Demographic information and cytokine/chemokine levels were compared between the groups (Table 4). IP-10 was significantly higher in the ANLF group than in the NLF group. Furthermore, IP-10 had a positive correlation with ALT and AST concentrations (*r*_*s*_ = 0.546 and 0.644, respectively; *P* < 0.001) (Figure 2). No significant difference in the circulating cytokine and chemokine levels was found between patients with distinct HBeAg status or HBV DNA load.

## 4. Discussion

Patients infected with HBV may have different outcomes owing to the host's immune system. In some adults, HBV can be spontaneously cleared; however, some will undergo viral persistence and become immune-tolerant phase patients, immune active responders, or inactive carriers, wherein chronic infection and inflammation persist for a significant time, which will progress to liver failure or hepatoma [10]. Each phase of HBV infection stimulates distinct viral kinetics and host immune responses [11]. Chronic inflammation is an important factor that leads to the progression of HBV infection. Therefore, this study was aimed at exploring the specific effects of proinflammatory and anti-inflammatory cytokines, as well as chemokines, exerted during the progression of HBV infection, HBeAg seroconversion, and impairment of liver function.

Our results showed that higher circulating inflammatory cytokine IL-17 and lower IL-1*β* levels were beneficial for the HBV spontaneous clearance; and higher IL-17 levels were negatively associated with the probability of hepatoma development. Circulating IL-17 is primarily derived from helper T 17 (Th17) cells. Activated Th17 cells mainly secrete IL-17, IL-6, IL-21, and tumor necrosis factor (TNF)-*α*. These cells also induce chemokines, such as CXCL1, CXCL2/MIP-2*α*, and CXCL8/IL-8, to induce inflammatory reactions by recruiting neutrophils, macrophages, and lymphocytes in local tissue [12–14]. Several researchers have demonstrated that Th17 cells or IL-17 play an important role in autoimmune diseases, transplantation immunity, and chronic and acute infections caused by bacteria, parasites, fungi, and viruses [15–17]. However, the effects of IL-17 or Th17 cells on HBV infection remain controversial, and studies on the correlation between IL-17 and HBV spontaneous clearance are scarce. Until now, the inflammatory effects of IL-17 or Th17 cells on liver injury in CHB were thought to be confirmed. Several studies have found that Th17/IL-17 is simultaneously the fuel and flame of a sustained proinflammatory environment [18–20]. However, were IL-17 only play an inflammatory role in patients infected with HBV? Seetharam et al. [21] demonstrated the anti-HCV effects of Th17 cells and IL-17 in liver transplant recipients. Wang et al. [22] also demonstrated that IL-17 could effectively suppress HBV replication in a noncytopathic manner, which indicated one of the mechanisms to suppress HBV replication by IL-17. In our study, higher circulating IL-17 levels were more likely involved in the spontaneous clearance of HBV, which is supported by Wang et al. [22]. Similar phenomena have also been observed in other clinical studies on antiviral treatment in HBV-infected patients. Yu et al. [23] observed an increased peripheral Th17 cell and circulating IL-17 in patients with CHB treated with nucleos(t)ide analogs. Furthermore, Zhang et al. [24] and Feng et al. [25] showed that serum IL-17 can transiently increase during the early stage of entecavir or interferon-*α* treatment of patients with CHB. Therefore, the antiviral effect of increased IL-17 expression in patients with HBV infection should be paid closer attention.

The inflammatory cytokine IL-1*β* is an important pleiotropic cytokine involved in HBV infection. IL-1*β* is believed to promote the progression of chronic liver diseases [26, 27]. The present study obtained the same observations. However, Watashi et al. [28] found that pretreatment with IL-1*β* and TNF-*α* remarkably reduced host cell susceptibility to HBV infection. We suggest that the early or late phase of infection may be a factor that influences the effect of cytokines or chemokines on disease occurrence and progression.

In hepatoma, the levels of circulating cytokines and chemokines vary, which could be due to the complex immune status under the conditions of both HBV infection and tumor presence. Liao et al. [29] reported that IL-17 and its receptor are predictors of poor outcomes in hepatocellular carcinoma, whereas Du et al. [30] found no differences in serum IL-17 protein and mRNA levels between patients with CHB and with hepatoma. Our results are contrasting to those of other studies. In our study, the level of IL-17 in the hepatoma group was significantly lower than that in previous studies [30, 31]. We speculate that this may be due to the small sample size and tumor heterogeneity. IL-17 can inhibit the tumor pathogenesis via immune-mediated tumor rejection, and it appears to be a pleiotropic cytokine with possible pro- or antitumor effects, depending on tumor immunogenicity [32]. Therefore, further functional studies considering tumor heterogeneity may be useful in elucidating the distinct effects of IL-17 on HBV-related hepatoma.

IP-10, a potent chemoattractant of activated T cells, has been the focus of studies on chemokines involved in chronic inflammatory diseases. Animal studies by Lang et al. [33] and Kakimi et al. [34] described in detail the mechanisms underlying liver inflammation caused by intrahepatic recruitment of inflammatory cells (monocytes and T cells) orchestrated by chemokines (CXCL-9 and IP-10). Meanwhile, high levels of circulating IP-10 have been detected in CHB patients with active liver inflammation, as well as in patients with acute hepatitis B, but not in patients with other acute viral infections [35, 36]. In our study, all chemokines in the CHB group were drastically increased, especially IP-10, which caused severe inflammation in patients with HBV and reflected the host responses to the active virus infection. Furthermore, a positive correlation between circulating IP-10 and AST or ALT in CHB indicated that circulating IP-10 exerted a critical inflammatory role in liver injury during CHB, which was consistent with the results of Tan et al. [36].

Here, we also focused on the association of cytokines/chemokines with HBV DNA load, HBeAg seroconversion, and liver damage. Our results demonstrated that only IP-10 was positively correlated with liver damage, similar to a previous study [37]. However, the association of circulating cytokine and chemokine levels with HBV DNA load or HBeAg seroconversion in patients with CHB was not observed. Several studies have shown that serum cytokines and chemokines in CHB are related to HBeAg status, viral replication, and stage of liver disease [8, 38–40]; however, the correlation between circulating cytokine or chemokine levels and CHB clinical characteristics remains controversial. Gigarda et al. [41] showed that IP-10 levels were not associated with HBeAg seroconversion in HIV1-HBV coinfection following HBV-active antiretroviral therapy. Thus, to date, only the role of IP-10 in necroinflammation in CHB has been confirmed.

## 5. Conclusions

Inflammatory cytokines and chemokines may be a “double-edged sword” for HBV clearance and progression. Our results showed that IL-17 is a potential biomarker for predicting disease progression and that higher circulating IL-17 levels may contribute to HBV spontaneous clearance. Furthermore, increased IL-1*β* and IP-10 levels could induce chronic inflammation and strengthen the liver injury observed in CHB, in which IP-10 is a major inflammatory factor.

Nevertheless, further studies should be conducted to clarify the specific effects of cytokines and chemokines in HBV progression. Based on our results, we can further explore the roles of IL-17, IL-1*β*, and IP-10 in HBV spontaneous clearance and chronic progression.

## Figures and Tables

**Table 1:** Characteristics of the enrolled subjects.

Characteristics	CHB (*N* = 81)	SC (*N* = 70)	Hepatoma (*N* = 42)	Control (*N* = 29)	*P*
Age^a^	39.49 ± 9.41	42.47 ± 10.26	39.95 ± 11.13	42.52 ± 10.43	0.231
Sex (male/female)	53/28	50/20	27/15	18/11	0.764
HBsAg+^b^	81 (100.00%)	0 (0.00%)	42 (100.00%)	0 (0.00%)	<0.001
HBsAb+^b^	0 (0.00%)	70 (100.00%)	0 (0.00%)	29 (100.00%)	<0.001
HBeAg+^b^	56 (69.14%)	0 (0.00%)	9 (21.43%)	0 (0.00%)	<0.001
HBeAb+^b^	21 (25.93%)	0 (0.00%)	35 (83.33%)	0 (0.00%)	<0.001
HBcAb+^b^	81 (100.00%)	70 (100%)	42 (100.00%)	0 (0.00%)	<0.001
HBV DNA (IU/mL)^c^	3.59 × 10^6^ (2.14 × 10^5^–1.92 × 10^7^)	NA	1.00 × 10^3^ (1.00 × 10^3^–2.76 × 10^4^)	NA	<0.001
TB (*μ*mol/L)^c^	14.7 (12.0–19.5)	13.1 (10.2–16.8)	18.5 (11.9–25.1)	11.9 (8.6–15.3)	<0.001
DB (*μ*mol/L)^c^	5.1 (4.0–7.1)	3.9 (2.9–5.1)	8.0 (5.3–13.3)	3.5 (2.5–4.8)	<0.001
IB (*μ*mol/L)^c^	10.2 (7.6–13.1)	9.3 (7.2–11.8)	9.3 (6.1–13.9)	8.1 (6.1–10.9)	0.229
ALT (IU/L)^c^	61 (35–139)	21 (15–30)	59 (39–106)	20 (14–30)	<0.001
AST (IU/L)^c^	46 (27–78)	22 (17–26)	72 (36–137)	23 (20–28)	<0.001
ALP (IU/L)^c^	84 (66–109)	71 (61–84)	105 (82–166)	69 (58–82)	<0.001
GGT (IU/L)^c^	28 (12–59)	23 (17–41)	71 (39–161)	19 (11–28)	<0.001
TP (g/L)^a^	75.0 ± 5.4	70.8 ± 4.8	61.4 ± 9.7	74.7 ± 4.2	<0.001
ALB (g/L)^a^	45.3 ± 4.6	45.4 ± 2.9	33.9 ± 6.0	46.9 ± 2.6	<0.001
RBC (×10^12^/L)^a^	4.80 ± 0.79	4.88 ± 0.46	4.18 ± 0.79	NA	<0.001
Hb (g/L)^a^	146 ± 26	149 ± 14	128 ± 23	NA	<0.001
WBC (×10^9^/L)^a^	6.14 ± 1.53	6.40 ± 1.68	8.66 ± 5.41	NA	0.002
PLT (×10^9^/L)^a^	179 ± 59	184 ± 56	176 ± 113	NA	0.888
NLF/ANLF	35/46	70/0	12/30	29/0	<0.001

Note: Values reported as ^a^mean ± standard deviation, ^b^frequency (percentage), and ^c^median (interquartile) Abbreviations: ALB: Albumin; ALP: Alkaline phosphatase; ALT: Alanine aminotransferase; ANLF: Abnormal liver function; AST: Aspartate aminotransferase; CHB: Chronic hepatitis B; DB: Direct bilirubin; GGT: Gamma-glutamyl transferase; Hb: Hemoglobin; IB: Indirect bilirubin; NA: Not available; NLF: Normal liver function; PLT: Platelets; RBC: Red blood cell; SC: Spontaneous clearance; TB: Total bilirubin; TP: Total protein; WBC: White blood cell.

**Table 2:** Circulating cytokine and chemokine levels of the study groups.

Groups	Proinflammatory cytokines			Anti-inflammatory cytokine	Chemokines			
	IL-1*β*	IL-6	IL-17	IL-10	IL-8	IP-10	MCP-1	MIP-1*β*
CHB (*N* = 81)	3.69 (2.20–5.30)	15.68 (13.10–28.29)	46.22 (41.07–53.75)	4.86 (4.14–7.82)	49.77 (23.76–203.30)	2456.50 (1682.16–4016.28)	49.60 (34.82–70.83)	228.94 (146.91–452.52)
SC (*N* = 70)	0.95 (0.50–1.51)	16.88 (14.57–20.60)	90.62 (73.95–115.70)	0.40 (0.40–3.02)	33.28 (24.45–52.27)	921.18 (669.29–1514.12)	59.97 (48.94–81.59)	125.49 (92.67–185.55)
Hepatoma (*N* = 42)	0.48 (0.48–2.94)	16.93 (4.10–39.42)	0.57 (0.44–41.07)	0.89 (0.40–7.20)	32.86 (7.40–163.93)	444.35 (235.82–1493.47)	12.53 (7.48–52.13)	57.96 (25.19–169.35)
Control (*N* = 29)	1.13 (0.93–1.88)	5.96 (3.24–11.18)	28.47 (20.74–53.57)	2.57 (1.48–3.63)	30.37 (14.57–44.94)	925.62 (737.16–1286.39)	49.21 (40.92–65.86)	210.33 (70.33–287.92)
*P*	<0.001	<0.001	<0.001	<0.001	0.003	<0.001	<0.001	<0.001

Note: Values are reported as median (interquartile), pg/mL. Comparisons among groups were performed using the Kruskal-Wallis H test. Abbreviations: CHB: Chronic hepatitis B; IL: interleukin; SC: Spontaneous clearance; IP-10: Interferon-*γ*-inducible protein-10; MCP-1: Monocyte chemoattractant protein-1; MIP-1*β*: macrophage inflammatory protein-1*β*.

**Table 3:** Multivariate logistic regression analysis between cytokine/chemokine levels and chronic hepatitis B outcomes.

Variables	SC		Hepatoma	
	OR (95% CI)	*P*	OR (95% CI)	*P*
IL-1*β*	0.052 (0.005–0.534)	0.013	NA ^b^	
IL-6	– ^a^		– ^a^	
IL-17	1.468 (1.080–1.995)	0.014	0.933 (0.910, 0.957)	<0.001
IL-10	NA^b^		NA^b^	
IL-8	NA^b^		– ^a^	
IP-10	NA^b^		NA^b^	
MCP-1	NA^b^		NA^b^	
MIP-1*β*	NA^b^		NA^b^	

Note: ^a^Variable was not included in the multivariate analysis because of the lack of statistical significance in the univariate analysis. ^b^Variable was not included in the logistic regression equation based on the forward LR stepwise method. Abbreviations: CI: Confidence interval; IL: Interleukin; MCP-1: Monocyte chemoattractant protein-1; MIP-1*β*: Macrophage inflammatory protein-1*β*; OR: Odds ratio; SC: Spontaneous clearance.

**Table 4:** Circulating cytokine and chemokine levels in chronic hepatitis B patients with different clinical characteristics.

Groups	N	Age	Male	IL-1*β*	IL-6	IL-17	IL-10	IL-8	IP-10	MCP-1	MIP-1*β*
*HBeAg statuses*											
Negativity	25 (30.86%)	41.24 ± 11.27	20 (80.00%)	3.28 (2.01–5.30)	15.68 (13.10–25.71)	45.54 (41.42–52.38)	4.86 (4.26–11.43)	49.77 (22.01–234.39)	2730.17 (1987.72–5695.15)	46.18 (34.93–66.37)	238.39 (158.31–558.52)
Positivity	56 (69.14%)	38.71 ± 8.46	33 (58.93%)	3.81 (2.37–5.57)	15.68 (13.01–29.68)	47.59 (40.38–54.43)	4.86 (3.90–7.32)	50.27 (25.20–161.79)	2341.39 (1330.84–3707.99)	52.21 (34.56–74.35)	218.35 (137.65–415.40)
*P* value		0.267	0.065	0.484	0.744	0.416	0.681	0.959	0.065	0.419	0.533
*HBV DNA load*											
Low	17 (20.99%)	41.94 ± 9.08	16 (94.12%)	2.92 (2.08–4.46)	14.51 (12.93–23.22)	43.48 (38.66–53.41)	4.86 (4.38–6.83)	49.77 (26.36–228.16)	2227.33 (1608.40–3224.34)	46.58 (34.11–79.88)	202.52 (147.17–452.52)
High	64 (79.01%)	38.84 ± 9.46	37 (57.81%)	3.84 (2.38–5.60)	15.68 (13.35–29.68)	47.59 (41.58–53.75)	4.86 (3.90–7.82)	50.27 (23.65–207.87)	2580.68 (1753.78–4306.71)	49.70 (34.72–68.11)	233.67 (139.44–480.21)
*P* value		0.230	0.005	0.200	0.580	0.265	0.948	0.724	0.302	0.424	0.889
*Liver function*											
Normal	35 (43.21%)	39.57 ± 10.08	21 (60.00%)	3.75 (2.18–5.69)	16.01 (13.84–37.77)	47.59 (42.10–53.75)	4.50 (3.90–14.86)	52.98 (28.45–353.12)	1920.92 (1291.94–2880.56)	41.23 (34.41–67.16)	246.01 (169.28–375.22)
Abnormal	46 (56.79%)	39.43 ± 8.99	32 (69.57%)	3.52 (2.32–5.27)	15.34 (12.68–25.70)	46.22 (39.01–53.75)	4.86 (4.32–6.95)	49.53 (22.01–172.64)	2941.38 (2154.07–4723.25)	56.36 (36.17–75.48)	200.30 (119.40–532.82)
*P* value		0.949	0.370	0.886	0.258	0.554	0.896	0.230	0.006	0.098	0.321

Note: All cytokine and chemokine levels are reported as pg/mL. Data are expressed as frequency (percentage), mean ± standard deviation, or median (interquartile), where appropriate. Quantitative data analysis was performed using Mann–Whitney *U* test and Student's *t*-test. The *χ*^2^ test was used to analyze the categorical data.

## Data Availability

The datasets generated and/or analyzed in the present study are available from the corresponding author upon request.
